# ZAK negatively regulates RhoGDIβ-induced Rac1-mediated hypertrophic growth and cell migration

**DOI:** 10.1186/1423-0127-16-56

**Published:** 2009-06-18

**Authors:** Chih-Yang Huang, Li-Chiu Yang, Kuan-Yu Liu, I-Chang Chang, Pao-Hsin Liao, Janet Ing-Yuh Chou, Ming-Yung Chou, Wei-Wen Lin, Jaw-Ji Yang

**Affiliations:** 1 Graduate Institute of Chinese Medical Science, China Medical University, Taichung 404, Taiwan; 2 Graduate Institute of Basic Medical Science, China Medical University, Taichung 404, Taiwan; 3 Department of Health and Nutrition Biotechnology, Asia University, Taichung 413, Taiwan; 4 School of Dentistry, Chung-Shan Medical University, Taichung 402, Taiwan; 5 Institute of Medicine, Chung-Shan Medical University, Taichung 402, Taiwan; 6 Chung-Shan Medical University Hospital, Chung-Shan Medical University, Taichung 402, Taiwan; 7 Department of Orthopedics Surgery, Chung-Shan Medical University, Taichung 402, Taiwan; 8 Midwestern University Chicago College of Osteopathic Medicine, Chicago, USA; 9 Cardiovascular Center, Veterans General Hospital, Taichung 407, Taiwan

## Abstract

RhoGDIβ, a Rho GDP dissociation inhibitor, induced hypertrophic growth and cell migration in a cultured cardiomyoblast cell line, H9c2. We demonstrated that RhoGDIβ plays a previously undefined role in regulating Rac1 expression through transcription to induce hypertrophic growth and cell migration and that these functions are blocked by the expression of a dominant-negative form of Rac1. We also demonstrated that knockdown of RhoGDIβ expression by RNA interference blocked RhoGDIβ-induced Rac1 expression and cell migration. We demonstrated that the co-expression of ZAK and RhoGDIβ in cells resulted in an inhibition in the activity of ZAK to induce ANF expression. Knockdown of ZAK expression in ZAK-RhoGDIβ-expressing cells by ZAK-specific RNA interference restored the activities of RhoGDIβ.

## Background

The mitogen-activated protein kinase (MAPK) signaling pathway consists of the sequentially acting upstream kinases MAPK kinase kinase (MAP3K) and MAPK kinase (MAP2K), and the downstream MAPKs, p38MAPK, extracellular signal-regulated kinase (ERK1/2), and c-jun N-terminal kinase (JNK). The mixed lineage kinases are a family of serine/threonine kinases, all of which are classified as MAP3Ks. The seven mixed lineage kinases cloned over the past several years can be classified into three subfamilies based on domain organization and sequence similarity: the MLKs (MLK1–4), the dual leucine zipper-bearing kinases (DLK and LZK), and the zipper sterile-α-motif (SAM) kinases (ZAKα and ZAKβ) [1,2]. ZAK can activate the JNK pathway and the nuclear factor κB (NFκB) pathway [3], and it induces JNK activation through a dual phosphorylation kinase, JNKK2/MKK7 [4]. Overexpression of wild-type ZAK induced apoptosis in a hepatoma cell line [3], and a recent report indicated that ZAK expression in a rat cardiac cell line, H9c2, induced hypertrophic growth and re-expression of atrial natriuretic factor (ANF) [5]. ZAK also mediates TGF-β-induced cardiac hypertrophic growth via a novel TGF-β signaling pathway [6]. In our previous study [5], we showed that the leucine zipper of ZAK mediates homodimerization and promotes autophosphorylation and JNK activation.

We identified RhoGDIβ(Rho GDP dissociation inhibitor beta) as a ZAK effector. RhoGDIβ, also known as Ly-GDI or D4-GDI, belongs to a family of Rho GDP dissociation inhibitors, and it is thought to regulate the activity and localization of Rho family proteins [7-10]. The RhoGTPase family includes Rho, Rac, and Cdc42, which differentially regulate the actin cytoskeleton [11-13] and function as molecular switches in cellular signal transduction by alternating between an inactive GDP-bound form that is maintained in cytosolic complexes with GDIs and a GTP-bound form that usually associates with the plasma membrane and interacts with downstream target proteins therein [14,15]. RhoGTPases regulate the reorganization of the actin cytoskeleton and the integrity of the integrin-associated adhesion complexes [16]. Rho facilitates stress fiber and focal adhesion assembly, Rac regulates the formation of lamellipodia and membrane ruffles at the leading edge of migrating cells, and Cdc42 triggers filopodia at the cell periphery [17]. RhoGDIs regulate RhoGTPase activity by inhibiting GDP dissociation to keep RhoGTPases in an inactive state.

A recent study indicated that stimulation of T lymphocytes and myelomonocytic cells with phorbol esters leads to RhoGDIβ phosphorylation on serine/threonine residues [18], raising the question of whether RhoGDIβ is involved in a signal transduction pathway in these cells. Thus, RhoGDIβ may play numerous roles in the regulation of biological activities; however, many details of the regulatory roles of RhoGDIβ remain to be elucidated.

## Materials and methods

### Northern blot analysis

Trizol reagents (Life Technologies) were used to isolate total RNA from H9c2 cells transiently transfected with the recombinant RhoGDIβ plasmids or from cells stably expressing RhoGDIβ. Total RNA (20 μg) was separated on a formaldehyde agarose gel, transferred to a nylon filter, and then hybridized with a probe corresponding to the full-length Rac1 cDNA labeled using the NEBlot random labeling kit (New England BioLabs) in the presence of [α-^32^P]dCTP. The blot was washed with SSC/SDS solutions (Sodium Chloride, Sodium Citrate/SDS) before autoradiography. Ethidium bromide staining was used to check the integrity of all samples.

### Wound healing assay

H9c2 cells seeded on 10-cm plates were cultured to confluency. They were then scratched with a 200 μl pipette tip and further incubated in DMEM supplemented with 10% FBS. Images were taken at 24, 48, and 72 h with a Zeiss Axiovert 200 microscope. The Image-Pro image analysis system was used to measure the lesion area. The data were expressed as the percentage of recovery (WC%) using the equation: WC% = [1 - (wounded area at T_t_/wounded area at T_0_)] × 100%, where T_t _is the number of hours post-injury and T_0 _is the time of injury.

### Membrane and cytosol fractionation

H9c2 cells were cultured with 1 μg/ml doxycycline for 48 h and then treated with lysis buffer (20 mM Tris-HCl, pH 7.5, 100 mM NaCl, 5 mM EDTA, 2 mM PMSF, 1× protease inhibitor) at 4°C for 30 min. The samples were centrifuged at 500 × g at 4°C for 10 min, and the pellets were dissolved in lysis buffer plus 0.1% (w/v) Triton X-100 for the membrane fractions. The supernatants were re-centrifuged at 15,000 rpm at 4°C for 20 min, and the supernatants were saved as cytosolic fractions.

## Results

### Expression of RhoGDIβ induces hypertrophic growth via modulation of Rac1 expression in cardiac cells

**Figure 1:**
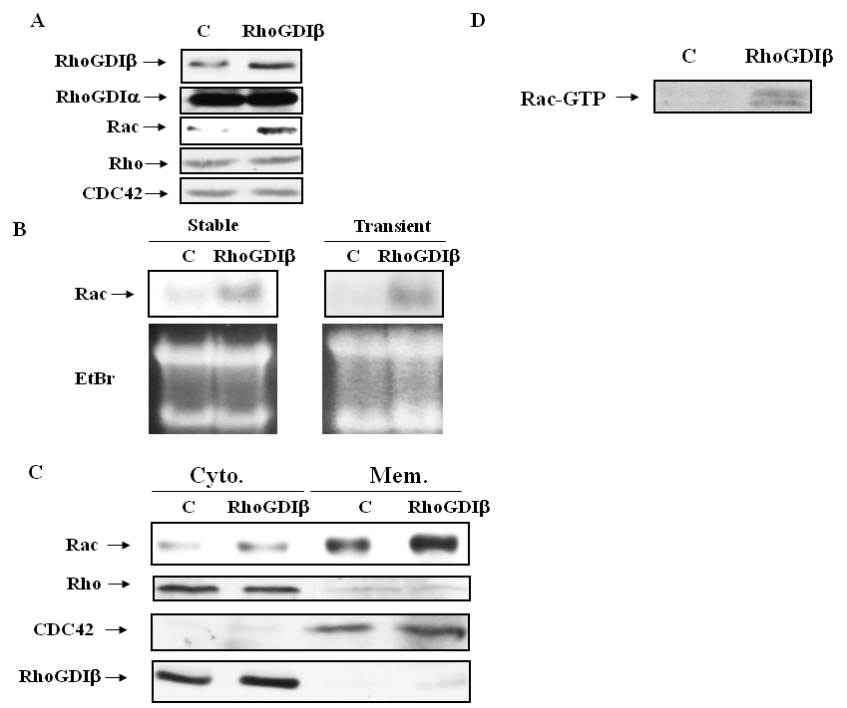
**Effects of RhoGDIβ on Rac1 expression and subcellular localization of RhoGTPases**. (A) RhoGDIβ, RhoGDIβ, Rho, CDC42, and Rac1 were detected by western blotting of cell lysates from H9c2 cells stably expressing RhoGDIβ. (B) Total RNA was isolated from H9c2 cells stably or transiently expressing RhoGDIβ. Rac1 transcripts were analyzed by northern blotting. The lower EtBr panels of (B) represent the 28S and 18S loading controls. (C) Membrane (Mem.) and cytosolic (Cyto.) fractions from H9c2 control (C) and RhoGDIβ-expressing cells were analyzed by immunoblotting for Rac1, Cdc42, and RhoGDIβ. (D) GTP loading in Rac1 was determined utilized PAK PBD binding assay in H9c2 cells stably expressing RhoGDIβ.

The RhoGTPases act as molecular switches by cycling between the inactive GDP-bound form located in the cytoplasm and an active membrane-associated GTP-bound form. The activities of Rho family proteins are regulated by various proteins, such as guanine nucleotide exchange factors (GEFs), GTPase activating proteins (GAPs), and GDIs. The functions and binding of RhoGDIα to RhoA, Rac1, and Cdc42 are well studied; however, the functions and targets for RhoGDIβ remain unclear, as it binds poorly to RhoA, Rac1, and Cdc42. We therefore sought to determine whether RhoGDIβ stimulates the expression or activities of these GTPases in cardiac cells by western blotting. The total RhoA and Cdc42 levels remained the same; however, cells overexpressing RhoGDIβ had increased levels of Rac1 (Figure 1A). Moreover, the expression of RhoGDIα in H9c2 cells was not affected by the overespression RhoGDIβ (Figure 1A). The expression levels of Rac1 may be causally linked to RhoGDIβ expression or merely an epiphenomenon of the selection of a stable clone. If the former is the case, then Rac1 might be a functionally important downstream target of RhoGDIβ. Northern blot analysis of total RNA indicated that cardiac cells either stably or transiently overexpressing RhoGDIβ had increased Rac1 mRNA levels (Figure 1B). We therefore concluded that RhoGDIβ plays a role in the transcriptional regulation of Rac1 and that the increased level of Rac1 mRNA was not a secondary effect of the selection of a stable RhoGDIβ-expressing clone. Rac1 association with membranes reflects its biological activity [19]. To further address the question of whether induction of Rac1 may also influence Rac1 activity, a detergent-insoluble membrane fraction was prepared from RhoGDIβ-overexpressing cells, and the levels of membrane-associated Rac1 were determined by western blotting. Both the levels of membrane-associated Rac1 and cytosolic Rac1 increased in RhoGDIβ-overexpressing cells (Figure 1C). Furthermore, the membrane-associated and cytosolic forms of Cdc42 remained unchanged in RhoGDIβ-expressing cells when compared to parental cells. We further detect the amount of GTP-bound Rac1 in H9c2 RhoGDIβ-overexpressing cells. We found that RhoGDIβ increased GTP loading in Rac1 (Figure 1D). Therefore, overexpression of RhoGDIβ in H9c2 cells increases the level of membrane-associated Rac1 and GTP loading in RAC1 by upregulating Rac1 transcripts. However, the increase in membrane-associated Rac1 in RhoGDIβ-expressing cells may be a secondary effect of increased expression of Rac1, as we were not able to detect any physical interaction between RhoGDIβ and Rac1 by co-immunoprecipitation.

**Figure 2:**
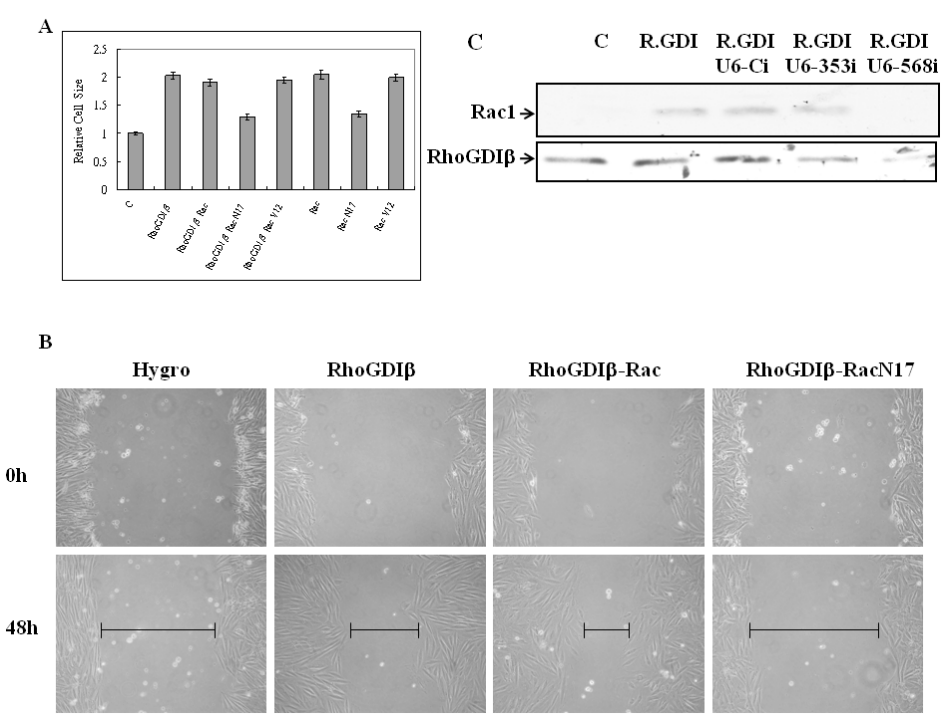
**RhoGDIβ-induced hypertrophic growth and cell migration is Rac1-dependent, but suppression of cell cycle progression is not**. (A) Control H9c2 and H9c2 RhoGDIβ-expressing cells stably transfected with Rac1, Rac1N17, or Rac1V12 were grown in 10% fetal bovine serum with doxycycline for three days, and the cell size was determined. (B) Wound-healing assay. The cell lines were seeded on plates, as previously described. After reaching confluency, the cell layer was wounded with a 200 μl pipette tip and incubated for 48 h with medium and doxycycline. (C) SiRNA knockdown of RhoGDIβ inhibited RhoGDIβ-induced Rac1 expression levels in H9c2 cells. U6-Ci is the scramble control siRNA, and both U6-353i and U6-568i are two specific RhoGDIβ siRNAs.

We next examined whether Rac1 mediates RhoGDIβ-induced hypertrophic growth. We found that H9c2 cells stably expressing a dominant-negative form of Rac1 (Rac1N17) and RhoGDIβ significantly reduced the twofold increase in cell size (Figure 2A) and actin organization induced by RhoGDIβ. Moreover, overexpression of wild-type or a constitutively active (V12) Rac1 in H9c2 cells was sufficient to induce hypertrophic growth (Figure 2A) and actin organization. These findings indicate that the RhoGDIβ-induced hypertrophic growth in H9c2 cells is mediated through increased expression of Rac1, and probably through increased levels of membrane-associated Rac1.

### H9c2 cell migration promoted by RhoGDIβ is Rac1 dependent

Rac1 is able to regulate cell migration [20]. To test whether the effect of RhoGDIβ on cell migration is Rac1 dependent, confluent monolayers of cells stably expressing RhoGDIβ, RhoGDIβ and Rac1, or RhoGDIβ and Rac1N17 were scrape-wounded with a sterile plastic pipette, and the migration of cells into the wound was monitored. The RhoGDIβ-expressing cells closed the wound area faster than control cells (Figure 2B). RhoGDIβ-expressing cells that also expressed a dominant-negative form of Rac1N17 migrated slower than RhoGDIβ-expressing or RhoGDIβ- and Rac1-expressing cells (Figure 2B), suggesting that Rac1 plays a key role in mediating cell migration in RhoGDIβ-expressing cells.

### RhoGDIβ-induced cell migration does not correlate with cell proliferation

To determine whether Rac1 might play a role in the RhoGDIβ-mediated cell cycle arrest, we examined the growth rate of cells expressing both RhoGDIβ and Rac1N17 and found that it was substantially slower than the growth rate of RhoGDIβ-expressing cells or control cells (Additional file 1). Therefore, the RhoGDIβ-regulated cell arrest was not mediated through increased levels of membrane-bound active Rac1.

Since the cyclin-dependent kinase inhibitors p21^Waf1/Cip1 ^and p27^Kip1 ^were expressed in RhoGDIβ-expressing cells at higher levels than they were in control cells, we examined p21^Waf1/Cip1 ^and p27^Kip1 ^levels in RhoGDIβ- and Rac1N17-expressing cells to study the effects of this dominant-negative form of Rac1 on the expression of p21 and p27. RacN17 was unable to block the expression of p21^Waf1/Cip1 ^and p27^Kip1 ^induced by RhoGDIβ in H9c2 cardiac cells (Additional file 2), suggesting that the RhoGDIβ-induced cell cycle arrest was not mediated through Rac1.

### Knockdown of overexpressed RhoGDIβ by siRNA reduces H9c2 cell migration

To this point, our results indicated that RhoGDIβ stimulates cell migration through the induction of Rac1 expression due to a proportional increase in the activity of Rac1 in H9c2 cells. To confirm the role of RhoGDIβ in cell migration, two RhoGDIβ knockdown cell lines were used to assess whether RhoGDIβ directly stimulates Rac1 expression to induce cell migration. The specific knockdown of RhoGDIβ using siRNA in H9c2 cells was confirmed by immunoblotting (Fig. 2C). We found that targeted disruption of RhoGDIβ by siRNA effectively blocked expression of Rac1 (Fig. 2C); therefore, RhoGDIβ depletion is associated with Rac1 downregulation. We used migration assays to confirm the role of RhoGDIβ in cell migration. Cells were seeded in an upper chamber of a Transwell on a porous filter, and the migration through the filter pores of H9c2 cells expressing RhoGDIβ and cells expressing both RhoGDIβ and RhoGDIβ- specific siRNA was compared. RhoGDIβ-expressing cells showed increased migration compared to parental cells, whereas migration was inhibited in the siRNA RhoGDIβ knockdown cells relative to the RhoGDIβ-expressing cells (Additional file 3). These results suggest that RhoGDIβ may play a critical role in the regulation of Rac1 expression and H9c2 cell migration.

### RhoGDIβ-induced wound healing is negatively regulated by ZAK

**Figure 3:**
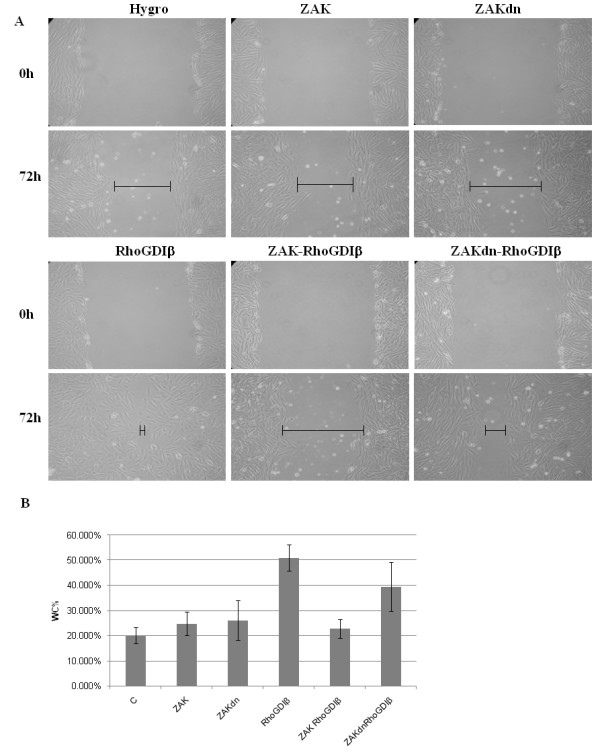
**ZAK reverses the effects of RhoGDIβ on the induction of cell migration in H9c2 cells**. (A) Wound healing assay. H9c2 cells and H9c2 cells ectopically expressing ZAK, dominant-negative ZAK (ZAKdn), RhoGDIβ, ZAK and RhoGDIβ, or ZAKdn and RhoGDIβ were seeded onto plates. After reaching confluency, the cell layer was wounded with a 200 μl pipette tip and incubated for 72 h with medium and doxycycline. (B) The cell migration capacity at 36 h was estimated by measuring the percentage wound closure (WC%). Values are means (SEM from three independent experiments).

Our experiments demonstrated that RhoGDIβ increased the rate of wound closure. However, our data also suggested that the physical association of ZAK with RhoGDIβ and phosphorylation of RhoGDIβ by ZAK could abolish RhoGDIβ function. To identify the regulatory role of ZAK on cell migration responses in RhoGDIβ-expressing cells, a wound-healing assay was performed using cells expressing RhoGDIβ, ZAK and RhoGDIβ, or ZAKdn and RhoGDIβ. After wounding, control, ZAK-expressing, and ZAKdn-expressing cells closed the gap at a similar rate (Figure 3A). As demonstrated above, RhoGDIβ-expressing cells were highly migratory, but ZAK- and RhoGDIβ-expressing cells were substantially slower to close the wound than ZAKdn- and RhoGDIβ-expressing cells. At 36 h post-wounding, control, ZAK-expressing, ZAKdn-expressing, and ZAK- and RhoGDIβ-expressing cells closed the gap by about 20%, 24%, 25%, and 22%, respectively (Figure 3B). RhoGDIβ-expressing cells and ZAKdn- and RhoGDIβ-expressing cells closed 50% and 39% of the gap. These data suggested a negative regulatory role for ZAK in RhoGDIβ cell migratory function.

**Figure 4:**
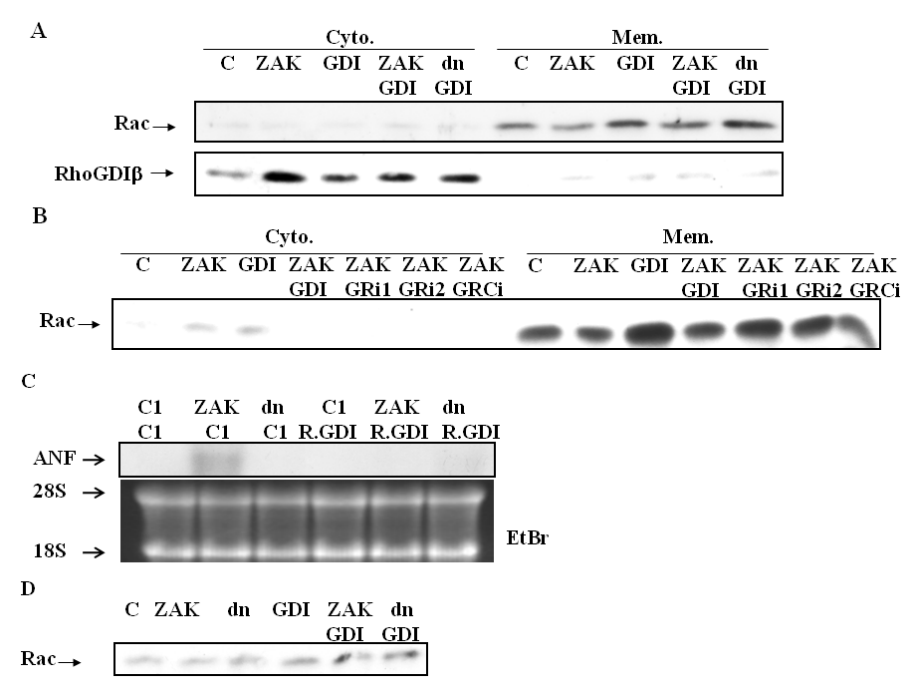
**ZAK specifically downregulates the activities of RhoGDIβ as a consequence of decreasing the amount of membrane-associated Rac1**. (A) ZAK decreases the levels of membrane-associated Rac1 induced by RhoGDIβ. Control H9c2 and transfected H9c2 cells were collected and fractionated into membrane (Mem.) and cytosolic (Cyto.) fractions by centrifugation. (B) SiRNA knockdown of ZAK restores the effects of RhoGDIβ in ZAK- and RhoGDIβ-expressing cells (GRi1 and GRi2) upon Rac1 membrane association. (C) RhoGDIβ inhibits ZAK functions upon the induction of ANF mRNA expression. (D) The effect of ZAK-RhoGDIβ and RhoGDIβ on total amount of Rac1expression.

To elucidate the role of RhoGDIβ in controlling the localization of Rac1, we performed confocal microscopy. The majority of Rac1 was present in the perinuclear region with some present at the plasma membrane of the control, ZAK-expressing, and ZAKdn-expressing cells (Additional file 4). More Rac1 was present at both the plasma membrane and the perinuclear region in RhoGDIβ-expressing cells. However, co-expression of ZAK, but not ZAKdn, with RhoGDIβ in H9c2 cardiac cells decreased the amount of Rac1 at the plasma membrane (Additional file 4). These data also suggested that ZAK might decrease the total amount of cellular Rac1, probably due to ZAK binding and phosphorylation of RhoGDIβ. To determine whether ZAK negatively regulates RhoGDIβ through expression of Rac1, especially membrane-associated Rac1, we performed western blotting. Consistent with the above experiment, membrane-associated Rac1 increased in RhoGDIβ-expressing cells, whereas, in ZAK- and RhoGDIβ-expressing cells, the levels of membrane-associated Rac1 decreased. However, co-expression of ZAKdn and RhoGDIβ had no such effect (Figure 4A). In this regard, it was of interest whether RhoGDIβ increases the amount of Rac1 at the plasma membrane as a consequence of increasing the total cellular levels of Rac1 or whether RhoGDIβ facilitates the translocation of Rac1 to the plasma membrane. We attempted to co-immunoprecipitate RhoGDIβ and Rac1to investigate this possibility; however, the antibody used was insufficient for this purpose (data not shown). This suggests that it is unlikely that RhoGDIβ facilitates translocation of Rac1 to the plasma membrane.

To test the importance of ZAK in regulating RhoGDIβ-mediated membrane-associated Rac1 and hypertrophic growth, we reduced the levels of ZAK using siRNA. We were able to reduce the levels of ZAK by expressing two different human ZAK siRNAs: ZAKGRi1 (ZAK-RhoGDIβ U6-460i) and ZAKGRi2 (ZAK-RhoGDIβ U6-1712i) (data not shown). The reduced levels of ZAK in these two individual clones were able to restore the levels of membrane-associated Rac1 to levels similar to those of RhoGDIβ-expressing cells, whereas the introduction of a scrambled ZAK siRNA (GRCi) into ZAK- and RhoGDIβ-expressing cells had no effect (Figure 4B). These results confirmed the importance of ZAK as a negative regulator of the effect of RhoGDIβ on the expression of Rac1 and membrane-associated Rac1. We found that ZAK was able to regulate ANF expression. We then studied the effects of RhoGDIβ on the regulation of ANF expression by ZAK by examining the levels of ANF mRNA in ZAK- and RhoGDIβ-expressing cells when compared with ZAK-expressing cells. The levels of ANF mRNA induced by ZAK were decreased in cells expressing both ZAK and RhoGDIβ (Fig. 4C). We also found that ZAK-RhoGDIβ cells has less total amount of Rac1 than RhoGDIβ cells. (Figure 4D). This result suggested that RhoGDIβ negatively regulates the functions of ZAK. Moreover, the data presented here (and consistent with our previous studies) indicate that both ZAK and RhoGDIβ may be hypertrophic growth inducers; however, ZAK physically interacts with RhoGDIβ and phosphorylates RhoGDIβ, thus inhibiting the ability of RhoGDIβ to induce Rac1 expression. The levels of Rac1 induced by RhoGDIβ are associated with the closure rate of wound healing (Figure 2B) and hypertrophic growth (Figure 2A), but they are not associated with cell cycle inhibition (Additional file 1). Thus, RhoGDIβ appears to play a role in signaling pathways regulating Rac1 expression that govern wound healing and hypertrophic growth in cardiac cells.

## Discussion

Upon introduction of RhoGDIβ into rat cardiac H9c2 cells, the cells exhibited hypertrophic growth, had a slower cell cycle, and migrated to a greater extent. We previously demonstrated that RhoGDIβ is phosphorylated by ZAK *in vitro*. It is striking that the co-expression of ZAK and RhoGDIβ in H9c2 cardiac cells results in the inhibition of the biological functions of RhoGDIβ, indicating that not only does RhoGDIβ possibly physically interact with ZAK, but it may also be negatively regulated by ZAK, and this regulation might occur via phosphorylation. These phenomena regulated by ZAK were correlated with Rac 1 expression and especially with the levels of membrane-associated Rac1 in H9c2 cells.

In H9c2 cells transiently and stably expressing RhoGDIβ, the levels of Rac1 transcripts increased compared with control cells. In this study, we described these surprising findings and, to our knowledge, the first demonstration that expression of RhoGDIβ induces Rac1 transcripts and increases the levels of membrane-associated Rac1. The results from western blotting and confocal microscopy experiments indicate that RhoGDIβ regulates Rac1 expression, which leads to increased levels of membrane-associated Rac1. We propose *either *that the increased levels of membrane-associated Rac1 in RhoGDIβ cells are merely a consequence of RhoGDIβ-induced expression of Rac1 *or *that RhoGDIβ regulates membrane translocation of Rac1. We were unable to detect an association between RhoGDIβ and Rac1 using co-immunoprecipitation; therefore, it is unlikely that RhoGDIβ andRac1 directly interact. However, we still have not ruled out the possibility that RhoGDIβ regulates Rac1 translocation. However, the signaling pathway between RhoGDIβ and Rac1 has not yet been elucidated, and there is currently no evidence that RhoGDIβ can directly bind to any gene promoter. RhoGDIβ can be translocated into the nucleus upon certain stimuli [21], leaving the possibility that RhoGDIβ can regulate gene expression directly. It is also possible that RhoGDIβ regulates Rac1 expression via signaling pathway effector proteins. Studies have also demonstrated that RhoGDIβ is cleaved at its N-terminus during apoptosis in a caspase-dependent manner and that the cleaved RhoGDIβ is retained in the nuclear compartment [22]. This suggests that RhoGDIβ could function in the nucleus.

We previously found that RhoGDIβ was able to associate with a mixed lineage kinase, ZAK, resulting in the phosphorylation of RhoGDIβ. To further study the role of ZAK in regulating the activities of RhoGDIβ, we used a bi-directional tet-on inducible system to express both ZAK and RhoGDIβ in H9c2 cardiac cells. Our results demonstrate that the levels of membrane-associated Rac1 and the hypertrophic growth phenotype were inhibited by co-expression of ZAK and RhoGDIβ; however, we did not observe the inhibitory effect with a dominant-negative form of ZAK. Clearly, the kinase activities of ZAK are necessary for the negative regulation of RhoGDIβ functions, including cell cycle arrest, hypertrophic growth, alterations in the amount of membrane-associated Rac1, and cell migration. Among all the biological functions that are regulated by RhoGDIβ, the phenomena of hypertrophic growth and cell migration are Rac1-dependent, whereas the regulation of the cell cycle arrest is Rac1-independent, as shown by the results of expression of a dominant-negative Rac1 (Rac1N17) in RhoGDIβ-expressing cells. It should be pointed out that the cell migration phenotype induced in RhoGDIβ-expressing cells seems to result primarily from the rate of migration rather than cell division. Therefore, RhoGDIβ may stimulate cell migration in a manner dissociated from its effects on cell cycle progression.

## Competing interests

The authors declare that they have no competing interests.

## Authors' contributions

CYH was responsible for the pull-down assay. LCY was responsible for the SiRNA knockdown. KYL was responsible for the cell migration assay. ICC, JIY, MYC and WWL were responsible for Western blot analysis. PHL was responsible for cell staining. JJY wrote the manuscript.

## Supplementary Material

Additional file 1 **Figure S1**. The growth rate of H9c2 cells expressing RhoGDIβ and Rac1N17 grown in 10% fetal bovine serum with doxycycline. Click here for file

Additional file 2 **Figure S2**. Expression levels of cyclin-dependent kinase inhibitors p21 and p27 in H9c2 RhoGDIβ-expressing cells and H9c2 cells expressing both RhoGDIβ and Rac1N17. Click here for file

Additional file 3 **Figure S3**. SiRNA knockdown of RhoGDIβ inhibited cell migration of H9c2 RhoGDIβ-expressing cells. Click here for file

Additional file 4 **Figure S4**. Confocal microscopy of H9c2 and H9c2 cells expressing the indicated proteins. Click here for file
