# Supplementary figures and images for: ZAK negatively regulates RhoGDIβ-induced Rac1-mediated hypertrophic growth and cell migration
Source: J Biomed Sci. 2009 Jun 18;16(1):56. doi: 10.1186/1423-0127-16-56 (PMC2703632; doi:10.1186/1423-0127-16-56)

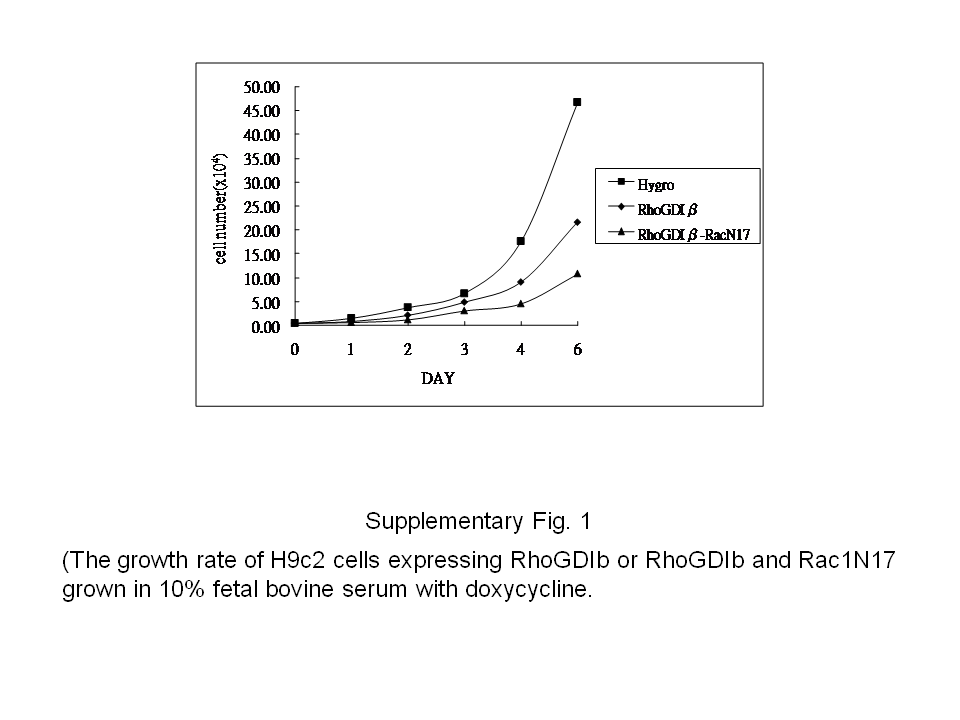

Supplement: Additional file 1 — Figure S1. The growth rate of H9c2 cells expressing RhoGDIβ and Rac1N17 grown in 10% fetal bovine serum with doxycycline. [file 1423-0127-16-56-S1.tiff]

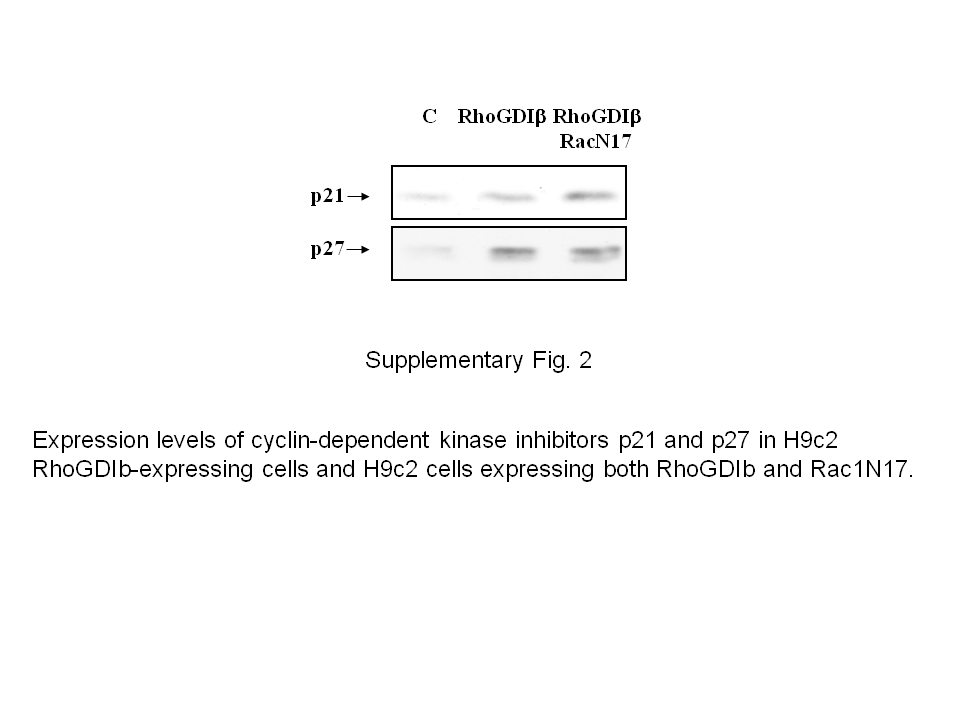

Supplement: Additional file 2 — Figure S2. Expression levels of cyclin-dependent kinase inhibitors p21 and p27 in H9c2 RhoGDIβ-expressing cells and H9c2 cells expressing both RhoGDIβ and Rac1N17. [file 1423-0127-16-56-S2.tiff]

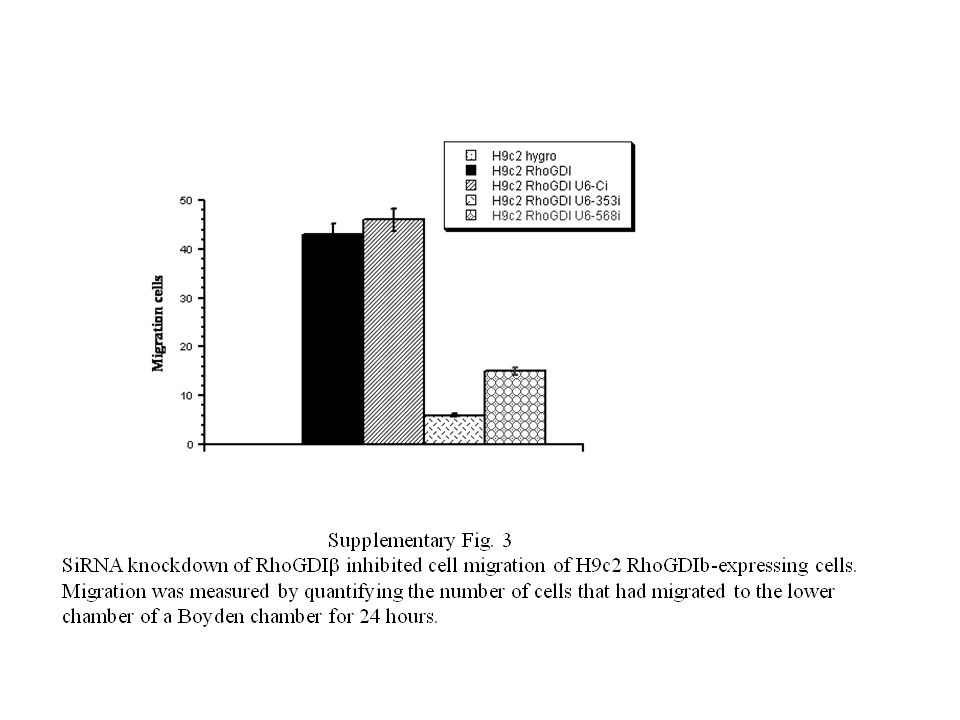

Supplement: Additional file 3 — Figure S3. SiRNA knockdown of RhoGDIβ inhibited cell migration of H9c2 RhoGDIβ-expressing cells. [file 1423-0127-16-56-S3.tiff]

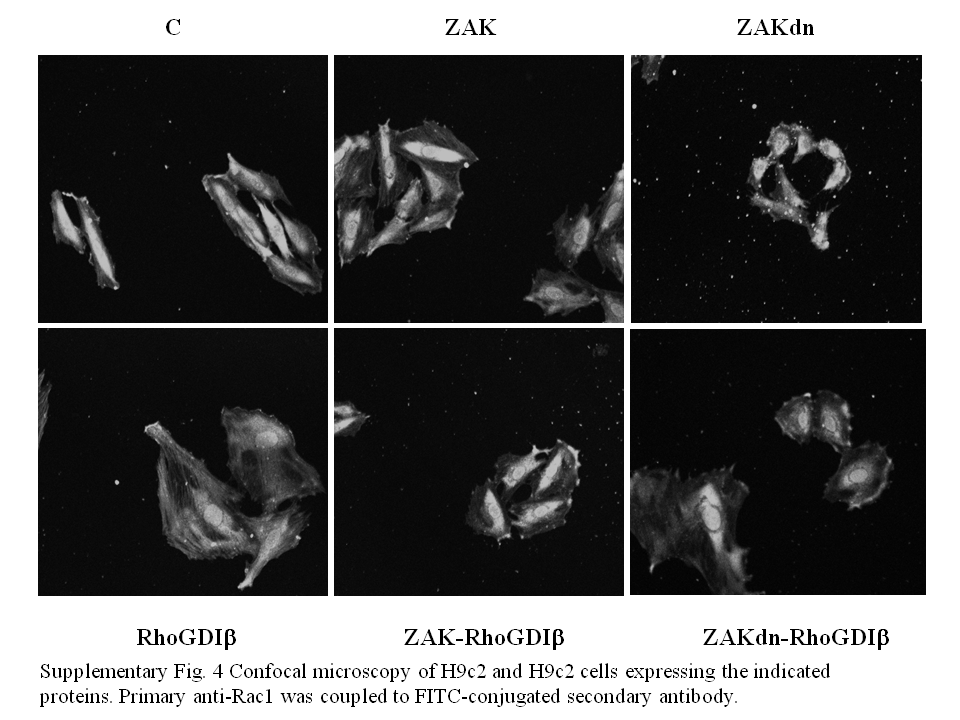

Supplement: Additional file 4 — Figure S4. Confocal microscopy of H9c2 and H9c2 cells expressing the indicated proteins. [file 1423-0127-16-56-S4.tiff]
